# Evaluating diurnal variations in retinal perfusion using optical coherence tomography angiography

**DOI:** 10.1186/s40942-020-00227-y

**Published:** 2020-06-03

**Authors:** Felix Rommel, Matthias Rothe, Maximilian Kurz, Michelle Prasuhn, Salvatore Grisanti, Mahdy Ranjbar

**Affiliations:** 1 Department of Ophthalmology, University of Luebeck, Ratzeburger Allee 160, 23538 Lübeck, Germany; 2 Laboratory for Angiogenesis & Ocular Cell Transplantation, University of Luebeck, Ratzeburger Allee 160, 23538 Lübeck, Germany

**Keywords:** OCTA, Retinal perfusion, Diurnal variation, Foveal avascular zone, Macular volume, Superficial plexus, Deep plexus, Full retina, Autoregulation

## Abstract

**Background:**

Optical coherence tomography angiography (OCTA) is a non-invasive tool for imaging and quantifying the retinal and choroidal vasculature as well as perfusion state in healthy eyes. Choroidal perfusion is subject to diurnal variation, showing lowest perfusion in the morning and highest in the afternoon. In this index study, OCTA was used to investigate diurnal changes of the retinal perfusion in healthy adult eyes and to identify impacting factors since retinal perfusion is known to be mainly determined by autoregulatory mechanisms.

**Methods:**

A prospective study was conducted on healthy volunteers, each of whom underwent repeated measurements of mean arterial pressure (MAP), intraocular pressure (IOP), macular volume (MV), subfoveal choroidal thickness (SFCT), foveal avascular zone (FAZ) and retinal perfusion of the superficial capillary plexus (SCP), deep capillary plexus (DCP) and full retina (FR) slab at 7 a.m. and 4 p.m. Possible influence of MAP or IOP on the retinal perfusion was evaluated.

**Results:**

A total of 22 eyes of 22 participants (mean age 55.91 ± 14.84) were analysed. Significant diurnal changes from 7 a.m. to 4 p.m. were observed for MAP (p < 0.001) and SFCT (p = 0.017). The perfusion of SCP, DCP and FR as well as the size of the FAZ and the MV did not show significant fluctuation during the day. No significant correlation between MAP or IOP and retinal perfusion values were detectable.

**Conclusion:**

OCTA-based analysis of the retina in healthy adults demonstrated a steady perfusion of both plexus during the day, independently of changes in MAP. These findings support the theory of autoregulatory mechanisms of the retinal blood flow.

## Introduction

The microvascular network of the macula is affected in many diseases such as retinal vascular occlusion and diabetic retinopathy. The capillary density and the foveal avascular zone (FAZ) play an important role in central vision and are good indicators for the progression of retinal diseases such as the above mentioned [1–3]. Information on changes in the macular capillary plexus is also important for a better understanding of macular diseases, its pathogenesis and prognosis. During the last 50 years, fundus fluorescein angiography (FA) has been the most popular tool to evaluate the retinal capillary perfusion and to obtain FAZ measurements. However, FA requires intravenous administration of contrast agent with possible adverse reactions [4]. Hence, follow-up examinations to monitor and compare capillary perfusion and FAZ over time are difficult to obtain. With the recent development of OCTA the retinal vascular network can be assessed in vivo and in real time by creating slab-segmented angiograms [5].

Diurnal variations have previously been shown for intraocular pressure (IOP), axial length (AL) and subfoveal choroidal thickness (SFCT) [6, 7]. Recently, we demonstrated significant diurnal patterns in choroidal sublayer perfusion in healthy adults and eyes with epiretinal membrane in OCTA-based studies [8, 9]. Analyzation of choroidal sublayer perfusion in healthy adults showed significant diurnal variation in Sattler’s and Haller’s layer perfusion. The lowest perfusion state was observed in the morning, the highest in the afternoon. Since the choroidal circulation is mainly controlled by autonomic innervation and retinal blood flow is mainly determined by autoregulatory mechanisms and local factors, we aimed to evaluate diurnal variations of retinal perfusion and the size of the FAZ using OCTA [10, 11].

## Methods

Participants for this prospective observational study were recruited from the Department of Ophthalmology at the University of Lübeck. The study was approved by the institutional review board and was conducted in accordance with the Declaration of Helsinki. All subjects received detailed information about the study and written informed consent was obtained individually by each participant before enrolment. Any history of ocular or cardiovascular disease, antihypertensive drug use, as well as diabetes mellitus was defined as exclusion criteria. Ethnically all participants were Caucasian and they underwent a thorough examination including blood pressure (BP), refraction, best-corrected visual acuity (BCVA) in Snellen, IOP, AL, slit-lamp biomicroscopy, macular SD-OCT as well as OCTA. The maximum permissible spherical and cylindrical aberration was ± 3 and ± 1 diopters, respectively.

Imaging was performed on all subjects without prior pupil dilatation using the HS-100 (Canon, Tokyo, Japan) OCT/OCTA device at 7 a.m. and 4 p.m. by a single, trained operator. Each imaging session included OCT (10 × 10 mm^2^) and OCTA (3 × 3 mm^2^) volumetric scans of the posterior pole. The devices’ follow-up mode was used to assure measurements at the same location for both time points. The HS-100 device works with a modified full-spectrum amplitude decorrelation algorithm to generate flow maps. Only OCTA scans with a signal strength ≥ 7, centered on the fovea, and the absence of motion as well as segmentation and projection artifacts were considered [12, 13].

Macular volume (MV) was acquired for both time points according to the Early Treatment Diabetic Retinopathy Study (ETDRS) grid, which contains three concentric rings of diameters 1, 3 and 6 mm around the fovea and two reticules to divide the macula into nine sections [14]. SFCT was measured manually just below the fovea, extending perpendicularly from hyperreflective Bruch’s membrane to the inner scleral border.

**Fig. 1:**
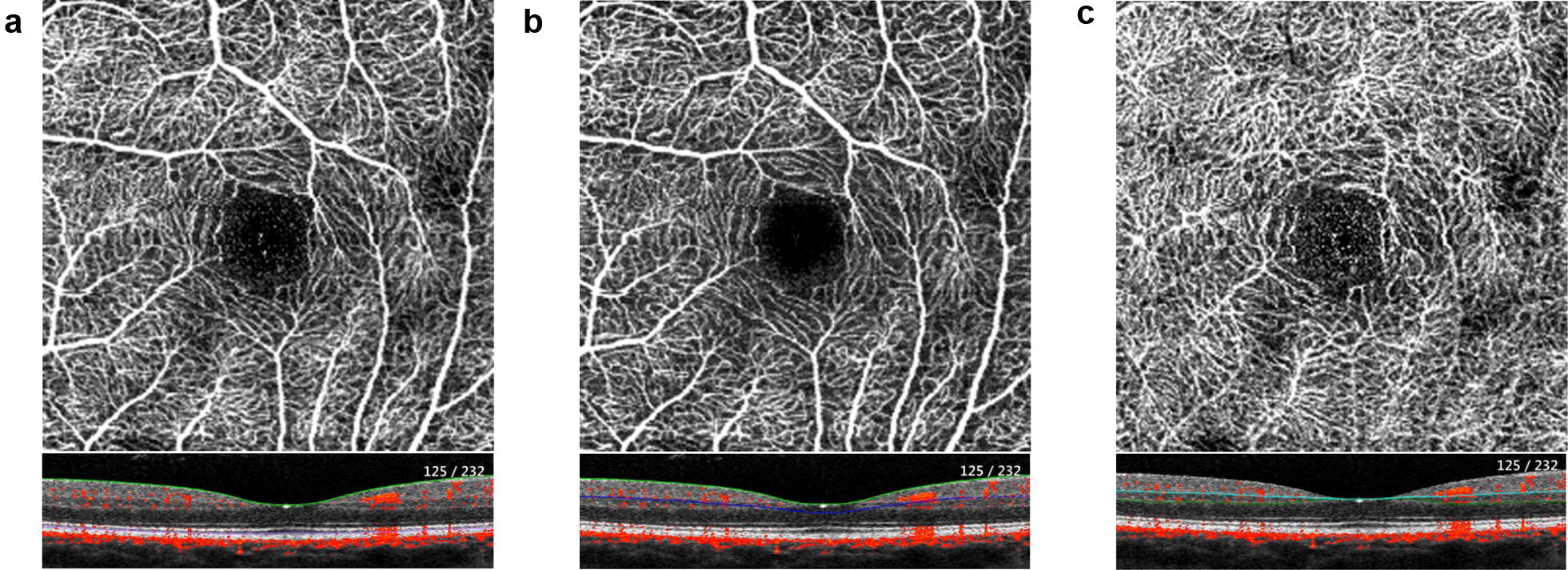
OCTA-Imaging of the posterior pole of a healthy adult. Angiogram and corresponding B-scan of the full retina (**a**), the superficial capillary plexus (**b**) and the deep capillary plexus (**c**)

After acquisition, OCTA images were automatically segmented in all B-Scans according to the manufacturer’s default setting to produce en face images of the superficial capillary plexus (SCP), deep capillary plexus (DCP) as well as a full retina (FR) slab (Fig. 1). The FAZ area (mm^2^) was manually measured in the FR slab by two experienced graders (F.R. and M.Ro.) and the mean value was used for statistical analysis.

Each en face image was exported into ImageJ (NIH, Version 1.48b, Bethesda, USA) and binarized by the Otsu method, which is an automatic threshold selection from grey-level histograms, to determine the percentage of white and black pixels [15]. Retinal perfusion was calculated by scoring the percentage of white pixels in relation to number of total pixels, according to published protocols [8, 9, 16].

Statistical analyses were performed using IBM SPSS (Version 24.0, Chicago, IL, USA) and Prism GraphPad (Version 8.0, La Jolla, CA, USA). BCVA measurements in decimal Snellen were converted to logarithm of the minimum angle of resolution (logMAR). Mean arterial pressure (MAP) was calculated based on systolic and diastolic BP (2/3 diastolic BP + 1/3 systolic BP). The Shapiro–Wilk test was used to check for normality of all obtained data. Diurnal changes in MAP, IOP, MV, SFCT, FAZ, SCP-, DCP- and FR-Perfusion were evaluated using Wilcoxon signed-rank test. The influence of changes in MAP, IOP and MV on macular perfusion was analyzed by Spearman’s correlation analysis. Inter-rater agreement between the two graders measuring the FAZ area was evaluated using concordance correlation coefficient (CCC) along with its 95% confidence interval (CI).

## Results

A total of 22 eyes of 22 healthy participants were recruited and included in the analysis. Demographic and clinical data are reported in Table 1. Laterality was assigned by chance, leading to 10 right eyes and 12 left eyes.

**Table 1:** Demographic and clinical data

Parameter	Mean ± SD	Median (min; max)
Age (years)	55.91 ± 14.84	59.5 (28; 76)
Sex (F/M)	12 (54.5%)/10 (45.5%)	
Axial length (mm)	23.83 ± 1.38	24.11 (21.83; 26.63)
BCVA (logMAR)	0.04 ± 0.05	0.00 (0.00; 0.10)

*F* female, *M* male, *SD* standard deviation, *BCVA* best-corrected visual acuity

Table 2 outlines the diurnal variations of the collected data. MAP showed significant diurnal changes, increasing from the morning (94.09 ± 5.01 mmHg) to the afternoon (99.91 ± 7.44 mmHg) measurement (p < 0.001). SFCT revealed statistically significant changes during the course of the day as well, by dropping from 337 ± 79.46 µm to 318.18 ± 74.57 µm (p = 0.017). IOP stayed steady from 7 a.m. (14.7 ± 3.0 mmHg) to 4 p.m. (14.5 ± 3.0 mmHg, p = 0.61). Likewise, MV did not show any significant fluctuation (8.61 ± 0.33 mm^3^ vs. 8.64 ± 0.33 mm^3^, p = 0.283). Inter-rater CCCs of FAZ area measurements were 0.995 (95% CI 0.988 to 0.998) at 7 a.m and 0.996 (95% CI 0.991 to 0.999) at 4 p.m., respectively. The mean measured size of the FAZ was 0.339 ± 0.119 mm^2^ in the morning and 0.336 ± 0.114 mm^2^ in the afternoon and didn’t show any statistically significant difference (p = 0.514).

**Table 2:** Diurnal changes in mean arterial pressure (MAP), intraocular pressure (IOP), macular volume (MV), foveal avascular zone (FAZ), superficial capillary plexus perfusion (SCP-P), deep capillary plexus perfusion (DCP-P), full-retina perfusion (FR-P), and subfoveal choroidal thickness (SFCT) are shown

Parameter	7 a.m. (Mean ± SD)	4 p.m. (Mean ± SD)	Wilcoxon signed-rank (p-value)
MAP (mmHg)	94.09 ± 5.01	99.91 ± 7.44	*< 0.001*
IOP (mmHg)	14.7 ± 3.0	14.5 ± 3.0	0.610
MV (mm^3^)	8.61 ± 0.33	8.64 ± 0.33	0.283
FAZ (mm^2^)	0.339 ± 0.119	0.336 ± 0.114	0.514
SCP-P (%)	29.16 ± 3.86	30.89 ± 5.99	0.189
DCP-P (%)	40.43 ± 3.93	40.98 ± 3.99	0.661
FR-P (%)	33.46 ± 4.67	35.08 ± 5.83	0.223
SFCT (µm)	337 ± 79.46	318.18 ± 74.57	*0.017*

Test values of p < 0.05 were considered statistically significant

**Fig. 2:**
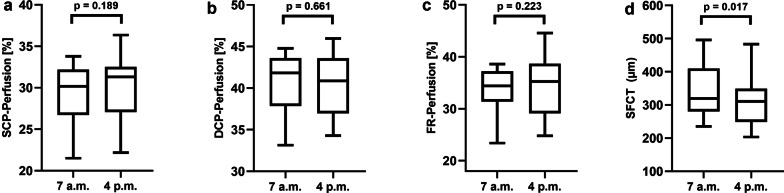
Boxplot analysis of the retinal sublayer perfusion and subfoveal choroidal thickness in healthy adults at 7 a.m. and 4 p.m. The perfusion in SCP (**a**) and DCP (**b**), as well as the full retinal perfusion (**c**) didn’t show statistically significant changes between 7 a.m. and 4 p.m., while SFCT significantly decreased. Test values of p < 0.05 were considered statistically significant

Perfusion measurements of the retina slabs didn’t reveal any significant diurnal fluctuations either (Fig. 2). Both, SCP- and DCP perfusion, showed a slight but not significant increase from 7 a.m. to 4 p.m. Perfusion in SCP went from 29.16 ± 3.86% in the morning to 30.89 ± 5.99% in the afternoon (p = 0.189) while DCP perfusion went from 40.43 ± 3.93% to 40.98 ± 3.99% (p = 0.661). Accordingly, perfusion of the FR slab did not show significant diurnal variation between 7 a.m. and 4 p.m. (33.46 ± 4.67% vs. 35.08 ± 5.83%, p = 0.223).

Further analysis did not reveal any significant correlation between diurnal changes of MAP and perfusion changes of SCP (r = − 0.32; p = 0.142), DCP (r = 0.04; p = 0.873) or FR (r = − 0.15; p = 0.497). Likewise, changes of IOP during the day did not significantly influence the perfusion of SCP (r = 0.13; p = 0.564), DCP (r = − 0.14; p = 0.528) or FR (r = − 0.02; p = 0.923).

## Discussion

To the best of our knowledge, this study is the first to evaluate diurnal changes in retinal perfusion by OCTA in healthy adults. Neither the perfusion in FR nor in SCP and DCP showed significant diurnal patterns between 7 a.m. and 4 p.m. However, our results of a significant decrease in SFCT during the day are consistent with previous studies regarding diurnal changes in SFCT [7, 18]. Although MAP significantly increased from morning to afternoon, the retinal perfusion stayed steady during the day. These results differ from findings of diurnal variation in choroidal perfusion. The healthy choroid, in particular Sattler’s and Haller’s layer, is known to be subject to diurnal variation [8, 17, 18]. Our recent OCTA-based study demonstrated a quadratic relation of the perfusion in both sublayers to time of the day, with the lowest perfusion state at 7 a.m. and the highest at 4 p.m. [8]. Several studies demonstrated the dependence of choroidal blood flow on MAP and even IOP due to poor autoregulation [8, 19, 20]. The present study indicates the independence of the retinal blood flow on changes of systemic blood pressure due to autoregulatory mechanisms. While choroidal blood flow underlays diurnal variation, retinal blood flow seems to stay steady during the day. Previous studies using laser Doppler velocimetry have already demonstrated, that changes in systemic perfusion pressure have only a negligible influence on retinal blood flow [10, 11, 21, 22]. The responsible mechanism for the insensitivity to systemic changes in perfusion pressure within the retinal circulation seems to be the absence of neuronal innervation in retinal vascular beds in contrast to the choroid. While histological studies have revealed a rich supply of autonomic vasoactive innervation for the choroid, the nerves do not go further into the retina [23, 24]. Therefore retinal blood flow is mainly under autoregulation by both myogenic and local metabolic mechanisms [25, 26]. The exact mechanisms are still unclear and focus of research. This study supports the theory of a steady retinal blood flow due to autoregulation in contrast to the choroidal blood flow with a diurnal pattern.

In contrast to our data, Müller et al. showed diurnal fluctuation of the macular flow density in the DCP in primary open-angle glaucoma (POAG) patients with a slight increase during the day [27]. The perfusion in SCP did not change significantly, consistent with our findings. Interestingly, they found a positive correlation between the flow density in SCP and MAP. These results indicate that patients with POAG may have reduced autoregulatory capacity of the retinal vascular network. This hypothesis is supported by the study of Evans et al. who compared the changes in retrobulbar ocular blood flow in POAG patients with healthy controls during supine and upright posture [28]. They conclude that glaucoma patients exhibit vascular autoregulatory abnormalities in the vessels distal to the central retinal artery.

Our study demonstrates a constant size of the FAZ during the course of the day, which is an important finding for studies dealing with FAZ measurements. OCTA has been reported as sensitive regarding alterations of the FAZ size and shape, which deals as an important parameter of retinal capillary integrity [3, 29, 30]. This biomarker might even have prognostic significance as enlargement of the FAZ, which has been found in ischemic diseases such as retinal vein occlusion or diabetic retinopathy, is associated with poor visual outcome [3, 31]. Since the size of the FAZ in healthy adults seems to stay steady during the day, it is not important to account for time of the day when comparing longitudinal OCTA data. Our mean measured FAZ size of 0.339 ± 0.119 mm^2^ at 7 a.m. and 0.336 ± 0.114 mm^2^ at 4 p.m. are comparable to previously published data [5, 32–34]. Furthermore, inter-rater agreement of the two graders measuring the FAZ dimensions was very high with CCCs of 0.995 at 7 a.m. and 0.996 at 4 p.m., respectively. These results indicate optimal inter-rater repeatability for manual FAZ measurements in 3 × 3 mm^2^ OCTA scans and they are consistent with previous reported high CCCs [35, 36]. OCT scans of the macula did not show significant changes in MV between 7 a.m. and 4 p.m. in our healthy study population. Several studies reported diurnal variations in macular thickness or MV in macular disease patients such as retinal vein occlusions and diabetic retinopathy [37–39]. The proposed mechanisms include the effect of gravity and hydrostatic pressure, nocturnal hypotension and changes in retinal metabolism. There are only limited studies in literature dealing with diurnal changes of MV in healthy adults. Ashraf and Nowroozzadeh examined MV in SD-OCT for three different time points [40]. They found a slight diurnal variation for the nasal and inferior ETDRS subfield with a greater MV at 7 a.m. than at 7 p.m. However, as in our study, they did not detect diurnal changes in total MV of healthy retinas. Jo et al. didn’t detect any significant diurnal variation in MV either by measuring at two different time points using SD-OCT [41]. In conclusion, the intact blood-retinal barrier, in contrast to macular diseases with an edema, may resist diurnal hydrostatic changes which lead to a steady MV.

The present study has some limitations. As we only examined participants at 7 a.m. and 4 p.m., we may have missed out on important information of the retinal perfusion at other times. The scanned area of 3 × 3 mm^2^ only represents a small, but very important part of the retina. A wider range of examination area may provide higher meaningfulness. Furthermore, the manual measurement of the FAZ size may represent a potential bias. To reduce this possible confounding factor, all measurements were performed in a masked fashion by two experienced graders and the average values were used for statistical analysis. In addition, our methodical approach is restricted by using a single OCTA device since perfusion values differ from device to device, depending on hardware, segmentation and software algorithms. Finally, the sample size represents a potential limiting factor leading to a mainly exploratory data analysis. To corroborate our findings, further studies with a larger number of participants will be necessary.

## Conclusion

In conclusion, OCTA is becoming an important non-invasive tool for imaging and quantifying the retinal vasculature and perfusion state. The present study is the first to evaluate diurnal changes in retinal perfusion by using OCTA in healthy adults. The full retinal perfusion, as well as the sublayer perfusion in SCP and DCP stayed steady during the course of the day although MAP showed significant fluctuations. These findings support the theory of autoregulatory mechanisms and local metabolites to control the retinal blood flow, independently of changes in systemic BP.

## Data Availability

The datasets used and analyzed during the current study are available from the corresponding author on reasonable request.

## Abbreviations

AL: Axial length
BCVA: Best-corrected visual acuity
BP: Blood pressure
DCP: Deep capillary plexus
ETDRS: Early Treatment Diabetic Retinopathy Study
FA: Fluorescein angiography
FAZ: Foveal avascular zone
FR: Full-retina
IOP: Intraocular pressure
MAP: Mean arterial pressure
MV: Macular volume
OCT: Optical coherence tomography
OCTA: Optical coherence tomography angiography
POAG: Primary open-angle glaucoma
SCP: Superficial capillary plexus
